# Deep Learning Based on Hierarchical Self-Attention for Finance Distress Prediction Incorporating Text

**DOI:** 10.1155/2021/1165296

**Published:** 2021-12-10

**Authors:** Sumei Ruan, Xusheng Sun, Ruanxingchen Yao, Wei Li

**Affiliations:** ^1^School of Finance, Anhui University of Finance and Economics, Bengbu 233030, China; ^2^School of Business, Xi'an Jiaotong-Liverpool University, Suzhou 215123, China

## Abstract

To detect comprehensive clues and provide more accurate forecasting in the early stage of financial distress, in addition to financial indicators, digitalization of lengthy but indispensable textual disclosure, such as Management Discussion and Analysis (MD&A), has been emphasized by researchers. However, most studies divide the long text into words and count words to treat the text as word count vectors, bringing massive invalid information but ignoring meaningful contexts. Aiming to efficiently represent the text of large size, an end-to-end neural networks model based on hierarchical self-attention is proposed in this study after the state-of-the-art pretrained model is introduced for text embedding including contexts. The proposed model has two notable characteristics. First, the hierarchical self-attention only affords the essential content with high weights in word-level and sentence-level and automatically neglects lots of information that has no business with risk prediction, which is suitable for extracting effective parts of the large-scale text. Second, after fine-tuning, the word embedding adapts the specific contexts of samples and conveys the original text expression more accurately without excessive manual operations. Experiments confirm that the addition of text improves the accuracy of financial distress forecasting and the proposed model outperforms benchmark models better at AUC and *F*2-score. For visualization, the elements in the weight matrix of hierarchical self-attention act as scalers to estimate the importance of each word and sentence. In this way, the “red-flag” statement that implies financial risk is figured out and highlighted in the original text, providing effective references for decision-makers.

## 1. Introduction

Financial distress is a global issue of significant concern for all stakeholders. It usually brings a tremendous amount of loss to the related parties [1, 2], which is a severe threat to the stability of global economic systems [3]. Due to loss avoidance, cost saving, and risk management, financial distress prediction is emphasized by potential investors, managers, government officials, and other decision-makers [4]. A scientific and informed prediction model is urgently in need.

Financial distress prediction is a typical binary classification. Most previous researches focused on the application of machine learning methods to gain insights into financial indicators as clues to detect financial risk. For model construction, on one hand, classic statistical and machine learning methods are applied in feature engineering and classification, such as Naïve Bayesian [5, 6], Support Vector Machine (SVM) [2, 7, 8], and ensemble learning including decision trees based Gradient Boosting Decision Tree (GBDT) [9–12], Random Forest (RF) [13, 14], eXtreme Gradient Boosting (XGB) [13, 15], and Adaptive Boosting (AdaBoost) [16, 17]. On the other hand, various deep learning models are also employed for modeling [18], such as Genetic Algorithm (GA) [6, 19], Convolutional Neural Network (CNN) [20, 21], and Self Organizing Map (SOM) [22]. In short, various models are used to exploit the risk information represented by limited financial ratios to forecast financial distress. This type of research has been quite sufficient.

Financial ratios are calculated in accordance with a specific framework, which provides an opportunity for the company to whitewash the financial situation within a limited range [22]. For example, financially distressed firms tend to undertake more accrual earnings management and less real earnings management [23, 24]. More essentially, forecasting simply covering financial indicators neglects the economic environment and recent business decisions reflected in other disclosure. In summary, the information conveyed by financial data is limited; it is still a challenging task to forecast financial risk accurately.

With the development of artificial intelligence (AI), experts in the field of finance and accounting devote themselves to integrating heterogeneous massive amounts of information by the devices with powerful computing capabilities to predict financial distress more accurately [12, 14, 17]. Relevant research proved that text fusion benefits more accurate identification of financial distress [4, 5, 25]. Since all listed companies obey structural rules to disclose annual reports, the majority of textual information is similar to each other except MD&A. MD&A is closely related to financial distress prediction as it offers investors the review of the company's performance as well as the future potential from the perspective of management [14, 25–27]. Thus, it is reasonable to extract texts from MD&A to represent the nonfinancial information for a supplement. However, the changeable semantic information and unstructured wordy content in MD&A are serious obstacles for text presentation.

There are already some paradigms to quantify text. Most related studies utilize bag-of-words method for text representation [5, 13, 14, 25, 28]. It means that these studies regard the text as a set of scattered segments or isolated words, counting all the terms according to the dictionary to represent text as word count vectors. However, it ignores the contexts hidden inside words and sentences. On the contrary, word embedding through designed neural networks (or pretrained neural networks) preserves the integrity of the article and makes it available to transform the contexts in the corpus into numeric tensors [26, 29, 30]. Compared with training the text embedding neural network based on certain own datasets, the pretraining model with more complicated structures has been trained on a massive standard corpus, with more powerful text representation ability. On a specific natural language processing task, text embedding adaptive for a certain dataset is obtained after fine-tuning the pretrained model. However, in this area, there have been few studies employing advanced pretrained neural networks for end-to-end text representation about financial distress prediction. In this way, Bidirectional Encoder Representations from Transformer (BERT) is introduced for word embedding in the study.

After each word in the text is expressed as a word vector, another major challenge is that the long sequence of information is difficult to remember. In the previous researches on text classification, most researches [31, 32] regard the text as a sequence of words and regard the output from RNN and LSTM as the representation of the text. Generally, multiple hidden layers in RNN and LSTM are considered to record the contextual information, which is summarized by the output of the last hidden layer. However, for lengthy text information, due to gradient diffusion and gradient explosion, this model tends to forget the previous information in the article. In comparison, attention is better in the classification of long-sequence texts [33, 34]. Only critical information where more weights are assigned is extracted. Although attention does not consider the order of words in the text, it is compensated by the text embedding expressed by the pretrained model, through which the position of each word is recorded.

Aiming to efficiently express the MD&A of large size and provide additional clues to detect financial distress, hierarchical attention neural networks (HAN) are proposed in this study. Since the length of MD&A is usually more than 1000 Chinese words, it is unrealistic to process the entire text as a tedious sentence. We draw on related research on the classification of hierarchical levels, split long texts into sentences, extract the main points of each sentence through attention, and express the sentence vector through the average word vector. On the basis of sentence vectors, the key sentence information is once again refined into text vectors by attention. In this way, the main points of the entire text are effectively expressed in the text vector. This text classification design is especially suitable for the processing of the lengthy MD&A. Based on a combination of original texts and financial ratios, comprehensive experiments have proved that the proposed model outperforms other baseline models trained on word count vectors or financial indicators at AUC and *F*2-score.

Our main contributions for financial distress prediction are demonstrated as follows:

For the prediction model, after word embedding, a framework based on hierarchical self-attention neural networks is proposed, competent for the binary classification of texts of large size. Contextual information is embedded as high-dimensional tensors by BERT. Then, attention effectively extracts essential information hierarchically at the word level and the sentence level. Along with financial ratios, as the risk information in MD&A is more effectively and comprehensively extracted, the predictive power of financial distress is enhanced.

For decision support and risk early warning, in consideration of visualization and interpretation, the weights of the attention matrix act as scalers to estimate the importance of linguistic features both at the word and sentence levels. In an article or a sentence belonging to a sample suspected of risk, sentences and words with higher scores will be marked and highlighted as red-flag segments. The parameters learned by the attention network are regarded as the contextual commonality of financially distressed disclosure. For each sample input, this mechanism refines and labels keynotes about risk prediction, providing a direct reference for decision-makers.

## 2. Literature Review

There are different views on the definition of financial distress. Altman [35] first puts forward the multivariate discriminant analysis to establish a financial distress warning model and proposes the *Z*-score model to evaluate the possibility of corporate bankruptcy. Beaver [36] defines the default on preferred dividends, and default on debt as financial distress. Altman defines a financial dilemma as a legally bankrupt business. Deakin [37] recognizes only companies that have gone through financial distress, insolvency, or liquidation for the benefit of creditors are in financial distress. Carmichael [38] considers financial distress to be a disruption of obligations in the form of illiquidity, insufficient equity, debt arrears, or insufficient funds. For China's A-share stock market, Shanghai and Shenzhen stock exchanges announced on April 22, 1998, that they would specially treat (ST) stock transactions of listed companies with the abnormal financial state. It mainly refers to two cases: one is the net profit of the listed company audited negative for two consecutive fiscal years, and the other is the net asset per share audited below the face value of the stock in the most recent fiscal year. Usually, a listed company titled ST faces severe financial deterioration, as a sign of financial distress. China's definition of listed companies in financial distress puts weight on profitability before debt defaults, more cautiously.

Based on the indicators covered, the research on financial distress forecasting can be divided into two categories; there are two categories to construct prediction models. On one hand, financial information is simply transformed into financial ratios, and there are intensive studies based on machine learning for feature engineering and classification [10, 16, 20, 39–41]. However, financial statement fraud is frequently committed by cunningly revising financial ratios even legally [24]. Actually, the financial fraudulent activities occurring globally in the past two decades were estimated to amount up to $5.127 trillion, with associated losses increasing by 56% in the past ten years [26]. It is not convincing enough to adopt financial ratios simply to predict financial distress [23, 24]. On the other hand, more studies begin to focus on nonfinancial information incorporating financial ratios to predict the financial distress to reach higher accuracy. Nonfinancial information, mainly disclosed textual information, has proved to play an important role in financial distress prediction, such as letters to shareholders [28], MD&A [5, 14, 26, 27, 29], or sentiment from annual reports [4, 14, 26], as a supplement to financial numerical information represented by financial ratios only.

There have been methods to accomplish tasks incorporating texts represented by word count vectors. Peng et al. [27] analyze letters to shareholders to build a bag of words (BOW), count word vectors, and propose a scheme for financial distress prediction. Hajek and Henriques [5] deal with counted sentiment words with a random subspace method as an additional feature for financial distress forecasting. Further, word2vec is a comparatively advanced model based on the artificial neural network, which encodes each word as sequential embedded vectors where contexts are included [42]. To record the sequential information, RNN allows retaining the input sequence as contexts for each segment, which is widely applied for natural language processing (NLP). Long-Short Term Memory (LSTM) [43] is a special type of RNN, comprised of different gates determining corresponding information forgotten or updated and enabling long-term dependencies to be learned. Based on these techniques, Mai et al. [29] employ shallow layers of neural networks for text embedding and apply RNN for text classification. Besides, Du et al. [10] apply pretrained word2vec neural networks for word embeddings and employ models based on bidirectional LSTM (Bi-LSTM) for risk prediction. However, the longer the input sequence is accepted by the RNN, the more likely the training fails to remember the previous part of the article due to gradient vanishment or gradient explosion. Thus, Long-Short Time Memory (LSTM) has made improvements on the basis of RNN, which tries to capture more nonadjacent semantic information through the cell state of a text sequence. Although LSTM introduces a large number of parameters in exchange for more expression length, its expression effect on longer texts is still limited.

Besides, there are two approaches to integrate information derived from the disclosure text and quantitative finance ratios. The first way is to directly combine text and financial indicators in the data set [4, 5, 25, 26]. The latter one is similar to ensemble learning, which reprocesses the separately learned text information and financial information [29], not prevailing for fusing text in financial distress prediction.

## 3. Methodology

The objective of the study is to incorporate text representation and financial ratios to predict financial distress. Generally, financial ratios are structural data and require no excessive preprocessing. Comparatively, unstructured text parsed from annual reports demands to be cleaned and to be transformed into numeric tensors further.

The majority of MD&A exceed 1000 words. It is necessary to disassemble the article into sentences as time distributed series and then encode each part. However, even if the article is split into dozens of sentences, the memory length of convolutional neural networks (CNN, LSTM, etc.) is quite limited. Hence, this article proposed a prediction model based on the hierarchical self-attention after word embedding by the pretrained model, BERT. Composed of 12 encoders and decoders, BERT concludes the word sequences through positional embedding in each component.

**Figure 1:**
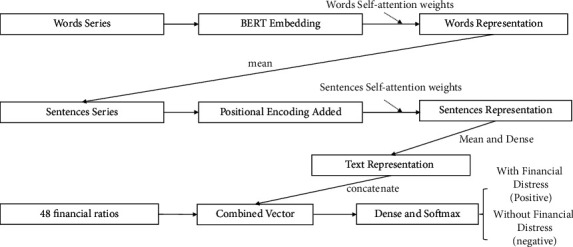
Flow chart of the proposed deep learning model.

The proposed hierarchical framework obtains the final text representation by averaging the sentence-level vectors when each sentence vector is the summary of the word vector. Self-attention treats the fragment most relevant to the other parts as significant information, as a typical efficient approach to deal with long sequences. Subsequently, financial ratios and dense text vectors are combined as final expressions, then identified by the fully connected layer as positive ones (with financial distress) and negative ones (without financial distress). The flow chart of the proposed method is demonstrated in Figure 1.

### 3.1. Hierarchical Attention for Text Representation

**Figure 2:**
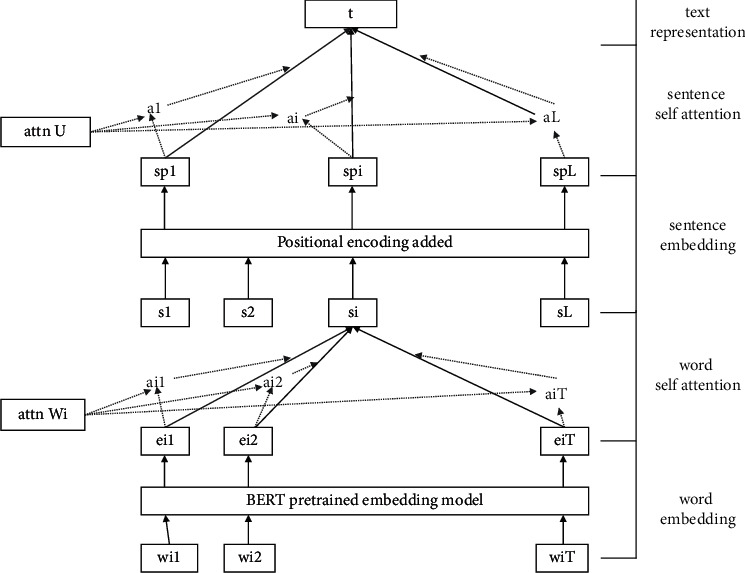
The architecture of hierarchical attention networks (HAN).

Hierarchical attention (HAN) for multilevel structures is an efficient framework for processing excessively long text information. The framework designed is inspired by Yang et al. [44]. On the one hand, the hierarchical construction divides the text with the large size into small pieces that can be accurately calculated. On the other hand, the model adapts the contexts of the same words or even the same sentences varying in different articles. Further, it endows each word or sentence specific expression according to certain contexts. The architecture of the hierarchical attention is shown in Figure 2.

#### 3.1.1. Word-Level Self-Attention

Here is the approach to obtaining sentence-level vectors from the word-level embeddings. The input was scattered isolated Chinese characters without extensive tokenization. *w*_*iτ*_ denotes the input character *τ* of the sentence *i*, *τ* ∈ [1, *T*], where *T* denotes the largest length of a sentence to be encoded.

Scaled dot-product is applied to generate self-attention. Weights in the values (*V*) are obtained by computing scaled dot-products of the query (*Q*) with all keys (*K*). In the word-level attention, the query denotes the embedding result of each word in the sentence  *i* embedded by BERT, *Q*_*i*_= [*e*_*i*1_,  *e*_*i*2_,…,*e*_*iT*_]^*T*^, and equals the key *K*_*i*_ and the value *V*_*i*_. The weights in square matrices *W*_*q*_,  *W*_*k*_,  *W*_*v*_ are parameters to be trained in the linear networks.

The element of the dot production matrix *W*_*i*_ measures the degree of similarity between two words in the word embedding space. *d*_*k*_ denotes embedding dimensions of words. It is assumed that *V*_*i*_^attn^ is the summary of sentence *i*, rewarding the keywords with more weights, while tending to neglect useless words with fewer weights.  *s*_*i*_ is the final sentence-level vector rerepresented by the mean of all word vectors in the word attention  *V*_*i*_^attn^.

(1) $$\begin{array}{c} Q_{i}=K_{i}=V_{i},\\ W_{i}=\text{softmax}\left(\frac{W_{q}Q_{i}\cdot W_{k}K_{i}^{T}}{\sqrt{d_{k}}}\right)=\left[a_{i1},a_{i2},\ldots ,\;a_{iT}\right],\\ V_{i}^{\text{attn}}=\left(Q_{i},K_{i},V_{i}\right)=W_{i}\cdot \;W_{v}V_{i}=\;{\left[e_{i1}^{\prime },e_{i2}^{\prime },\ldots ,e_{iT}^{\prime }\right]}^{T},\\ s_{i}=\frac{\sum_{\tau =1}^{T}e_{i\tau }^{\prime }}{T}. \end{array}$$

#### 3.1.2. Sentence-Level Self-Attention

The way to summarize sentence-level vectors as a final text vector is similar to how to refine word-level inputs from sentence-level input. The text sample *t* is composed of sentence queries *Q*_*t*_= [*s*_1_,  *s*_2_,…,*s*_*L*_]^*T*^, which equals keys *K*_*t*_ and values *V*_*t*_. The weights in square matrices *U*_*q*_, *U*_*k*_, *U*_*v*_ are parameters to be trained in the linear networks. The element in the dot production *U*_*t*_ measures the similarity between two sentences in the article. *d*_*s*_ denotes the embedding dimensions of sentences. It is considered that *V*_*t*_^attn^ denotes re-represented information contained in all the sentences of the document *t*. In this way, sentence-level attention assigns larger weights to the essential sentences. *t* is the final text vector represented by the mean of all the sentence-level vectors in the sentence attention matrix *V*_*t*_^attn^.

(2) $$\begin{array}{c} Q_{t}=K_{t}=V_{t},\\ U_{t}=\text{softmax}\left(\frac{U_{q}Q_{t}\cdot U_{k}K_{t}^{T}}{\sqrt{d_{s}}}\right)=\left[a_{1},a_{2},\ldots ,\;a_{L}\right],\\ V_{t}^{\text{attn}}=\left(Q_{t},K_{t},V_{t}\right)=U_{t}\cdot \;U_{v}V_{t}=\;{\left[s_{1}^{\prime },s_{2}^{\prime },\ldots ,s_{L}^{\prime }\right]}^{T},\\ t=\frac{\sum_{l=1}^{L}e_{l}^{\prime }}{L}. \end{array}$$

Subsequently, the model takes text vector generated from sentence-level representation as input to concatenate financial ratios.

### 3.2. Interpretation

After normalization by the soft-max function in rows, the element of the dot products in the symmetric matrix *W*_*i*_ scores the resemblance between word vectors in the sentence  *i*. If most words in a sentence resemble a certain word *w*_*t*_, the word is assumed to be the keyword. The sum of the elements in the column or row *i* of the matrix *W*_*i*_, ∑_*j*≠*t*_^*T*^*w*_*tj*_(*j*=1,2,…, *T*) , is regarded as the importance score to evaluate how often the word *w*_*t*_ is cited in the sentence  *i*. Notably, the element on the main diagonal is excluded from the evaluation.

(3) $$\begin{array}{c} {\text{imp}}_{i\tau }=\sum_{j\neq \tau }^{T}w_{\tau j}. \end{array}$$

Identically, the evaluation of the importance of each sentence in the article also follows the evaluation above. The sum of the elements in the column *s* in the matrix *U*_*s*_, ∑_*j*≠*s*_^*L*^*u*_*sj*_, (*j*=1,2,…, *T*) , is treated as the importance score to measure the frequency of the sentence *s* quoted by the other sentences.

(4) $$\begin{array}{c} {\text{imp}}_{s}=\;\sum_{j\neq s}^{L}u_{sj}. \end{array}$$

In order to discover significant sentences containing the main idea in one text, the importance score of the sentence is sorted and the top-ranked sentences with high imp_*s*_ should be concerned by deciders if the sample is labeled with financial distress. If the decision-makers would check the keywords of the red-flagged sentence  *i*, those words with excessive scores imp_*iτ*_ should be highlighted.

## 4. Experiment

The data set applied to the proposed model includes both texts of MD&A and financial indicators. Generally, there are two types of listed companies, including companies with special treatment (ST, positive samples) and normal companies (non-ST, negative samples). It is reasonable to mark listed companies to be titled ST or directly delisted as positive samples with financial distress one or two years ahead. Besides, the ratio of positive and negative samples of the original data set is 1 : 12. Financial distress prediction is challengeable with such a severely imbalanced dataset. Random undersampling is applied in this experiment. By reducing the number of negative samples, more features derived from positive samples can be noticed by the model.

The core mission is to combine the multisource of information for financial distress forecasting, where one of the difficulties is digitizing text information and combining text representation with financial ratios. The proposed model is compared with the baseline models with word count vector to represent text in the comparative experiments. Besides, in order to present the benefits of information fusion, experiments on financial data simply are also carried out.

Here are details on the implementation of the trial. For the device, the type of graphics processing unit (GPU) applied in this study is NVIDIA TITAN XP. In the process of processing text, the number of batch training takes a value of 4 with the epoch of 2. For the parameter fine-tuning, the hierarchical learning rate is also adopted, 2 × 10^−5^ is still proven to be the best learning rate for the pretrained model, and the learning rate of the custom networks is 0.001. With the dropout ratio of text encoding increasing slightly, the recall of positive samples has been effectively improved with acceptable precision.

Besides, 10-fold cross-validation is employed to make sure that there is no violent fluctuation for the generalization performance under the set of hyperparameters.  Section 4.3 shows the average of measurements under all the data divisions.

### 4.1. Data

The data in this experiment includes two parts, financial indicators and text MD&A. The text and numeric ratios are directly combined in one data set.

After all, the samples with financial distress are extremely few. In this study, there are 860 positive samples and 11140 negative samples in the original data set listed in Table 1. The ratio of positive samples (with financial distress) to negative samples is 1 : 12. Financial ratios and textual disclosure are included in the research, derived from listed companies in Shanghai and Shenzhen Stock Exchange markets from January 2012 to December 2018.

#### 4.1.1. Imbalance Treatment

The effect of learners will decline with the severely unbalanced dataset [7, 10, 45]. It is necessary to preprocess the imbalanced train set. In this study, certain majority samples with negative labels are reduced based on the random undersampling technique (Rus). The final sample distribution is demonstrated in Table 2.

#### 4.1.2. Text Data

Annual reports of listed companies are downloaded from Chinese official information query station designated by the China Securities Regulatory Commission information, the earliest securities information professional website, covering more than 3700 listed companies in Shanghai and Shenzhen Stock Exchange markets.

Nonfinancial information, MD&A, is extracted from annual reports. Generally, in addition to the financial indicators calculated by the financial staff, MD&A shows management's expectations for the company's prospects. It is assumed that the narrative of the disclosure hints at the company's governance or development trend [5, 25, 27].

It is worth mentioning that, to prevent overfitting, all company names and geographic locations in documents are filtered by the stop words list. For linear models or decision tree-based models, the BOW is employed to quantify text. For the model proposed in this study, raw text without extensive processing is directly entered as the input. However, the size of the MD&A is excessively large, most of which are beyond 512 words, exceeding the maximum length of the naïve BERT. If all the text in one sample is regarded as a sentence truncated within 512 words, it means that some essential content would be dropped off. Hence, it is necessary to divide the text into hierarchical levels, sentences, and words, to intergrade more information. Due to the limitation of hardware, only 1000 characters or less at the beginning of the document are entered into the proposed model. Each text is staged into 20 sentences within 50 words.

#### 4.1.3. Quantitative Data

The quantitative financial indicators are downloaded from the China Stock Market and Accounting Study database (CSMAR). Based on previous researches [5, 10, 12, 24], 48 financial indicators are taken into account, including solvency, ratio structure, operation, profitability, cash flow, risk, development, and the index of per share. Solvency and cash flow describe a company's ability to repay short-term and long-term debts to prevent bankruptcy. The ratio structure shows the value composition of the company. Operation and profitability evaluate the company's operating efficiency and performance. Risk measures the multiple that a small change in revenue leads to a huge change in profit due to the existence of fixed costs. Development capability refers to the speed at which a company expands.

### 4.2. Metrics

Financial distress prediction is regarded as a binary classification. There are four predicted results, true positive (TP), false positive (FN), true negative (TN), and false negative (FN). Only TP denotes correct performances to identify samples with financial distress as positive, while FP denotes wrong performances to identify samples without financial distress as positive. Correspondingly, TN indicates correct performances to identify negative samples as negative, and FN denotes wrong performances to mistake positive samples for negative ones.

For the identification of financial distress, the recall of positive samples is crucial. In this study, the model performance is evaluated by a combination of metrics, including the AUC, precision score, recall rate, *F*1-score, and *F*2-score for positive samples. The *F*-score is a combination of precision (the ratio of true positive identified by the classifier to all the positive samples) and recall (the proportion of identified positive samples to all positive samples).

(5) $$\begin{array}{c} \text{precision}=\frac{\text{TP}}{\text{TP}+\text{FP}},\\ \text{recall}=\frac{\text{TP}}{\text{TP}+\text{FN}}. \end{array}$$

Thus, the *F*-score measures how accurate and prudent are those for classifier's performance. Craja et al. [26] estimate the cost of neglecting a positive sample with financial problems to be twice as high as the cost of mistaking a negative sample for a positive one. Effective models should concentrate on the higher recall of positive samples. It is natural to emphasize that recall is more crucial than precision in financial distress prediction. This study employs the *F*2-score as a supplement to the *F*1-score. Besides, the AUC evaluates the ability to rank positive samples and negative samples in the correct order [10], also serving as an indicator.

(6) $$\begin{array}{c} F1-\text{score }=\frac{2\times \text{Precision}\times \text{Recall}}{\text{Precision }+\text{ Recall}},\\ F2-\text{score }=\frac{\left(1+\beta^{2}\right)\times \text{Precision}\times \text{Recall}}{\beta^{2}\times \text{Precision }+\text{ Recall}}\left(\beta =2\right). \end{array}$$

### 4.3. Comparative Experiment Result

Multiple sets of comparative experiments are carried out in this part. Generally, there are two groups, models on financial data simply and models on the combination of financial ratios and digitization of texts. The result of experiments on financial data serves as a benchmark to demonstrate the progress of different learners after adding text features. Typical baseline learners, including linear models (LR, SVM), the decision-tree based models (XGB, RF, and AdaBoost), and Multilayer Perceptions (MLP) serve as comparative models.

The evaluation indicators of all learners' performance on different data set divisions are reported. Multiple sets of train sets and test sets are generated with several random seeds to reduce bias in case of overfitting on the specific splitting.

#### 4.3.1. Modeling on Financial Ratios

Based on 48 financial indicators, the learning result of control models is shown in Table 3. As mentioned above, in addition to AUC, what should be concentrated on are the indicators of the learner's recognition of positive samples, recall, and *F*2-score. For these indicators, decision-tree based models perform well with higher AUC, recall. Especially XGB outperforms the other models in terms of AUC, recall, and *F*2-score. Although linear models, LR and SVM, have achieved higher precision, they leave out excessive positive samples, fail to serve as qualified learners in this area. Besides, ANN is composed of two encoders. Each encoder includes two linear layers and a fully connected layer. From the results, the performance of ANN is close to linear learners.

#### 4.3.2. Modeling on Financial Ratios and Digitalization of Text

It is the core of this research to intergrade financial indicators and text to predict financial distress. Typical approaches to convert text include BOW and word embedding through neural networks. BOW counts the word frequency in each text according to the dictionary manipulated by chi-square test and pair-words merging. BOW serves as a baseline method. The combined numeric word frequency vector with financial ratios vector is entered into benchmark leaners.

As a comparison to BOW, with the pretrained model BERT to represent texts, the result of the comparison experiments is shown in Table 4.

After adding text features, the effects of all models have been improved, with the exception of RF. It is observed that all models have unanimously made progress on the most noteworthy *F*2-score. When focusing on the AUC and *F*2-score, the proposed model achieved the best results with 82.18% and 71.41%. It can be concluded that when the *F*2-score, which puts weights on the recall rate, is regarded as the core indicator of the financial distress prediction, the proposed model behaves best. When dealing with texts with intricate internal relationships of intact original documents, deep neural networks (DNN) offer substantial improvement in interpreting the complexity and detect more commonality shared by positive samples. Our proposed model, BERT + HAN, proves to be a promising alternative method with the performance under a higher recall, which is emphasized by stakeholders.

### 4.4. Interpretation Demonstration

According to assumptions, the documents disclosed by companies facing financial difficulties have a certain contextual commonality instead of the simple frequency of words. These sharing features are summarized, captured by the elaborately designed hierarchical attention mechanism.

**Figure 3:**
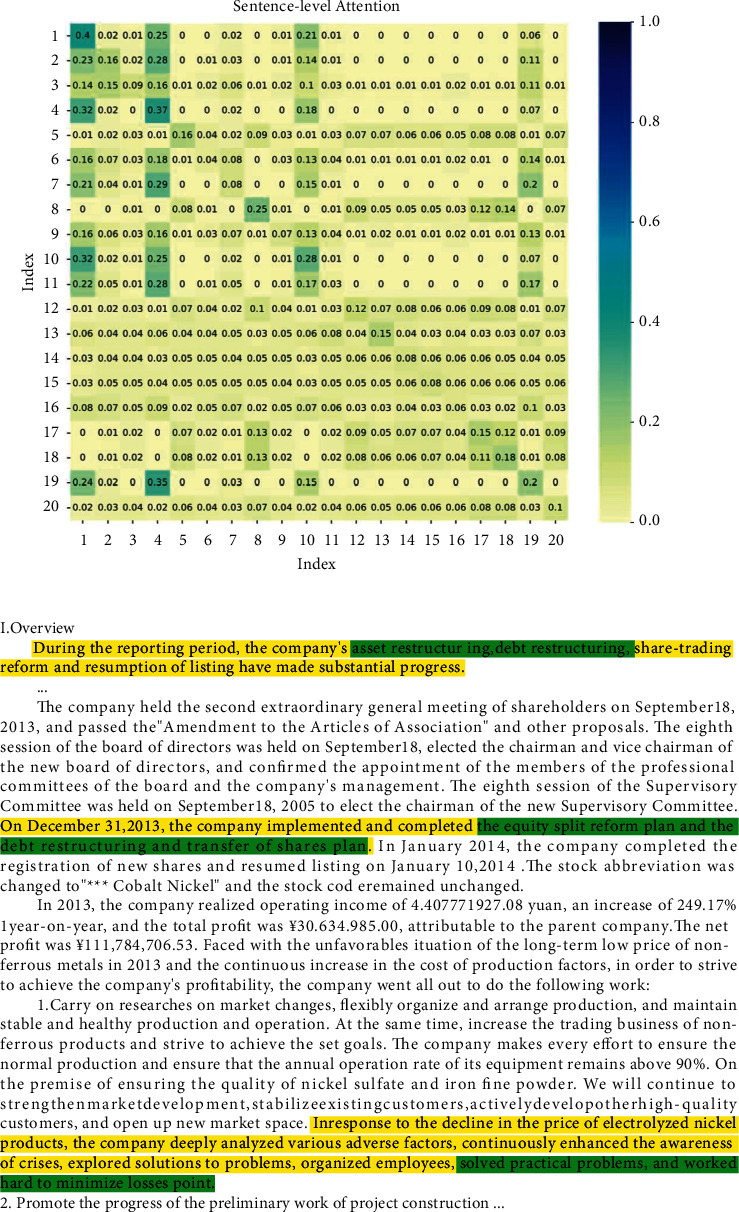
A page from MD&A parsed from a positive sample. In the sentence-level attention, corresponding to the top three total scores of column weights, the three sentences that best summarize the article information are highlighted. Similarly, the keywords in each sentence are also marked according to word-level attention respectively, where word-level attention is not depicted here.

Here, the identification of significant sentences and words in a sample facing financial distress is illustrated. In the text-level attention, each row of the matrix has been normalized. The sum of each column is considered to be the total cited score, in other words, the importance of the sentence of the column index. For the example illuminated in Figure 3, sentences with the serial number 1, 4, and 10 are evaluated and marked with the highest scores. In the same way, the keynotes in each sentence are also selected and highlighted with a darker color. The text-level attention and labeled article are displayed in Figure 3. Due to space limitations, the word-level attention matrix is not shown in the picture. Since the text is cleaned, and the sentences with the total number of words less than 50 are merged, the serial number corresponds to the cleaned text and may not correspond to the original sentence one-to-one.

The proposed model not only provides a more powerful financial distress prediction ability, but also the two-step attention mechanism offers an interpretable reference for decision-makers. Visual labeling of suspicious words and sentences offers clues to potential financial distress.

## 5. Discussion

Regarding the textual disclosure of new information as a supplement to financial indicators, a basic prerequisite is that it contains information that is not reflected in the latter, such as management's insights and expectations of the company's outlook. Moreover, companies facing financial distress have potentially similar contextual characteristics in disclosure, difficult to be modified like financial indicators. Our work confirms this, and through the setting of hierarchical attention networks, the exploration of the contextual features mentioned above has been well completed.

Our study introduces the pretrained model BERT with a powerful ability for text representation and employs a hierarchical attention mechanism to disassemble the ultra-long text into some shorter sentences for representation and training and, finally, combine the obtained text vector and financial data for financial distress prediction. From the experimental results, our proposed model beats all the benchmark models at the AUC and *F*2-score emphasized in the field. Experiments prove that the context of the original text hides clues to financial distress. If these clues are detected, they effectively improve the ability to predict financial distress.

To think further, the plain word2vec based on shallow neural networks and the bag-of-words perhaps have limitations in dealing with the text of large size, and it is difficult for them to capture the intricate and contextual attributes. With the original form of the text remaining, utilizing pretraining models BERT based on deep neural networks with fine-tuning and filtering the key information of long texts hierarchically based on the attention mechanism is a novel idea for analyzing large texts. More importantly, for different samples, attention is targeted to analyze and opt for indispensable features in varying contexts, which is closer to the way people process financial disclosure in reading comprehension. It is more effective than the methods quantifying text with one unified feature scale.

In addition, we have also explored the interpretability of deep neural network models. The attention mechanism provides a way to visualize the key features of all samples. Based on the vector similarity measurement by dot product normalized through soft-max function, we can pick up the key information and encode sentence vectors according to the word-level attention matrix and then refine the text vector through the sentence-level attention, where all the steps are visualized. Illuminating attention to different sentences and words and evaluating importance points, clues of financial distress in the original text can be marked.

We recommend that decision-makers pay more attention to the complex and tedious text disclosures. In particular, we expect that the proposed model can reduce the workload of auditors by filtering out key information. Through tracking and investigation of the clues further, the risk is more likely to be detected in advance.

## 6. Conclusion

Based on heterogeneous information, not only studies in the financial field to predict financial distress are involved, but also artificial intelligence methods to digitize unstructured information are necessary.

The model proposed in this research embeds and expresses the text from the original data at the word and sentence levels and summarizes the final vector representation of the text. Next, the text vector obtained and financial data are entered into the multilayer perceptron and classified. Experiments show that the proposed model beats all the benchmark ones at *F*2-score.

Without additional discretion, the potential of the proposed end-to-end deep learning method in information representation and feature engineering has been examined in this study. At the same time, the trained attention mechanism in this study successfully imitates humans to dig keynotes from complex language structures and offers readers with visualization of the “red flag” content as clues of financial distress. Finally, for researchers, research on the time series of corporate disclosure texts and financial indicators based on panel data may still be required. In addition, risk prediction divided by industry segments may be more effective in the application of artificial intelligence in the respective field.

## Figures and Tables

**Table 1:** The sample distribution of the original dataset.

Class	Number
Positive samples (titled “ST” in the next 2 years)	862
Negative samples	11142
Total samples	12004

**Table 2:** The sample distribution of the original dataset.

Class	Number
Positive samples (titled “ST” in the next 2 years)	862
Negative samples	2978
Total samples	3840

**Table 3:** Evaluation of models on 48 financial ratios.

		AUC	Precision	Recall	*F*1-score	*F*2-score
FIN	LR	0.6768	**0.8450**	0.372	0.5166	0.4189
	SVM	0.7506	0.7768	0.5465	0.6416	0.5809
	XGB	**0.8023**	0.7222	**0.6802**	**0.7006**	**0.6882**
	RF	0.7829	0.7448	0.6279	0.6814	0.6482
	ANN	0.7337	0.644	0.5581	0.5980	0.5734
	AdaBoost	0.7933	0.7604	0.6453	0.6981	0.6654

**Table 4:** Evaluation of models on both 48 financial ratios and text.

		AUC	Precision	Recall	*F*1-score	*F*2-score
FIN + BOW	LR	0.7203	0.8515	0.4826	0.6160	0.5284
	SVM	0.7729	**0.8683**	0.5258	0.6594	0.5708
	XGB	0.8115	0.7356	0.7035	0.7192	0.7097
	RF	0.7634	0.6357	0.6121	0.6237	0.6167
	ANN	0.7636	0.5720	0.6962	0.6280	0.6672
	AdaBoost	0.8071	0.7214	0.6860	**0.7061**	0.6986

FIN + TXT	**BERT** **+** **HAN**	**0.8218**	0.6656	**0.7274**	0.6951	**0.7141**

## Data Availability

1. The financial ratios data used to support the findings of this study have been deposited in the CSMAR repository (https://www.gtarsc.com/). 2. The annual reports data used to support the findings of this study have been deposited in the CNIFO repository (http://www.cninfo.com.cn/new/index).
